# A long pentraxin-3-derived pentapeptide for the therapy of FGF8b-driven steroid hormone-regulated cancers

**DOI:** 10.18632/oncotarget.3831

**Published:** 2015-04-14

**Authors:** Arianna Giacomini, Sara Matarazzo, Katiuscia Pagano, Laura Ragona, Sara Rezzola, Michela Corsini, Emanuela Di Salle, Marco Presta, Roberto Ronca

**Affiliations:** ^1^ Department of Molecular and Translational Medicine, University of Brescia, Brescia, Italy; ^2^ NMR Laboratory, Istituto per lo Studio delle Macromolecole, CNR, Milan, Italy

**Keywords:** angiogenesis, FGF8, hormone-regulated cancer, pentraxin, peptide

## Abstract

Fibroblast growth factor-8b (FGF8b) affects the epithelial/stromal compartments of steroid hormone-regulated tumors by exerting an autocrine activity on cancer cells and a paracrine pro-angiogenic function, thus contributing to tumor progression. The FGF8b/FGF receptor (FGFR) system may therefore represent a target for the treatment of steroid hormone-regulated tumors. The soluble pattern recognition receptor long pentraxin-3 (PTX3) binds various FGFs, including FGF2 and FGF8b, thus inhibiting the angiogenic and tumorigenic activity of androgen-regulated tumor cells. Nevertheless, the complex/proteinaceous structure of PTX3 hampers its pharmacological exploitation. In this context, the acetylated pentapeptide Ac-ARPCA-NH_2_ (ARPCA), corresponding to the *N*-terminal amino acid sequence PTX3(100-104), was identified as a minimal FGF2-binding peptide able to antagonize the biological activity of FGF2. Here, we demonstrate that ARPCA binds FGF8b and inhibits its capacity to form FGFR1-mediated ternary complexes with heparan sulphate proteoglycans. As a FGF8b antagonist, ARPCA inhibits FGFR1 activation and signalling in endothelial cells, hampering the angiogenic activity exerted *in vitro* and *in vivo* by FGF8b. Also, ARPCA suppresses the angiogenic and tumorigenic potential of prototypic androgen/FGF8b-dependent Shionogi 115 mammary carcinoma cells and of androgen/FGF8b/FGF2-dependent TRAMP-C2 prostate cancer cells. In conclusion, ARPCA represents a novel FGF8b antagonist with translational implications for the therapy of steroid hormone-regulated tumors.

## INTRODUCTION

Fibroblast growth factor-8 (FGF8), originally cloned from an androgen-dependent mouse mammary carcinoma cell line, belongs to the angiogenic FGF family [1, 2]. Like other members of the FGF family, FGF8 mediates its cellular responses by binding and activating tyrosine kinase FGF receptors (FGFRs) [3]. Experimental and clinical evidences point to an autocrine/paracrine role of FGF8 in the growth of epithelial/stromal cells in steroid hormone-regulated tumors [4, 5], the *FGF8* gene containing a functional androgen-response element responsible for its transcriptional activation by steroid-receptor signalling [6]. Alternatively spliced isoforms of the human *FGF8* gene allow the transcription of four different isoforms designated FGF8a, FGF8b, FGF8e, and FGF8f [7]. Among them, FGF8b is endowed with the strongest tumorigenic and angiogenic potential [4, 8]. Indeed, several studies demonstrate that FGF8b triggers angiogenesis, tumor growth and invasion in prostate cancer [5, 9]. Accordingly, FGF8b transgenic expression targeted to the prostate epithelium causes prostatic intraepithelial neoplasia (PIN) [10] whereas its downregulation inhibits the tumorigenic potential of prostate cancer cells [11]. Also, FGF8b was found to be highly expressed in breast and ovarian cancers [4, 8]. Thus FGF8b represents a possible druggable target for multidrug or multimodality treatment of steroid hormone-regulated tumors [4, 6].

The soluble pattern recognition receptor long pentraxin-3 (PTX3) is a member of the pentraxin family produced locally in response to inflammatory signals [12]. Previous observations had shown that PTX3 binds various FGFs *via* its *N*-terminal extension, including FGF2, FGF6, FGF8b, FGF10 and FGF17 [13-16], and inhibits FGF2-dependent angiogenic responses [16, 17]. Accordingly, transgenic PTX3 overexpression efficaciously impairs the activation and signaling of the FGF/FGFR system in FGF-driven tumor cell lines, thus affecting tumor growth and metastasis [15, 16, 18]. In particular, PTX3 inhibits the angiogenic and tumorigenic activity of androgen-regulated tumor cells in which testosterone activates a FGF8b-dependent autocrine/paracrine loop of stimulation [15].

PTX3 is a 340 kDa protein composed of eight 381 amino acid protomers [19]. The complex proteinaceous structure of PTX3 hampers its pharmacological exploitation. In this context, the 480 Da acetylated pentapeptide Ac-ARPCA-NH_2_ (in single letter code, hereafter referred to as ARPCA), corresponding to the *N*-terminal amino acid sequence PTX3(100-104), was identified as a minimal anti-angiogenic FGF2-binding peptide able to interfere with the biological activity of FGF2 [20]. Thus, ARPCA may represent an interesting FGF-trap molecule for the treatment of FGF-dependent tumors.

Here, we demonstrate the capacity of ARPCA to bind FGF8b, thus inhibiting its angiogenic activity *in vitro* and *in vivo*. Accordingly, ARPCA suppresses the angiogenic and tumorigenic potential of prototypic androgen/FGF8b-dependent Shionogi 115 (S115) mammary carcinoma cells [21] and of androgen/FGF8b/FGF2-dependent TRAMP-C2 prostate cancer cells [22]. Thus, ARPCA represents a novel FGF8b antagonist with possible implications for the therapy of steroid hormone-regulated tumors.

## RESULTS

### ARPCA binds and antagonizes FGF8b

**Figure 1:**
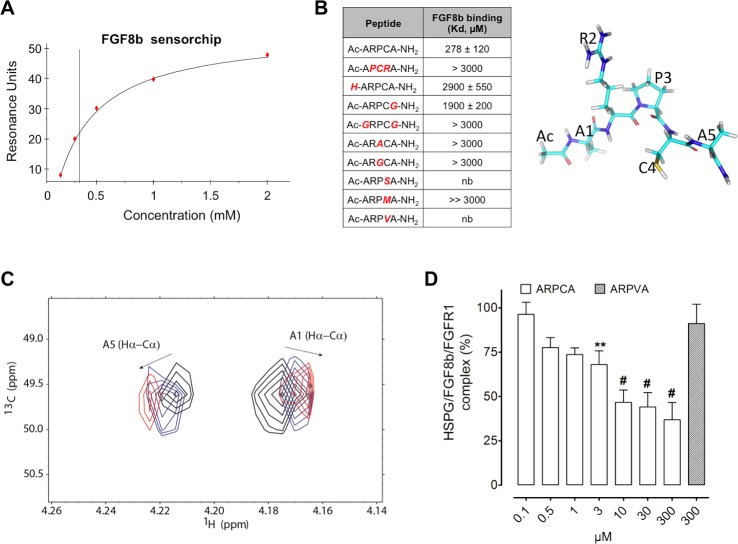
ARPCA binds and antagonizes FGF8b **A.** SPR analysis of ARPCA binding to immobilized FGF8b. **B.** Increasing concentrations of ARPCA and of peptide mutants were tested by SPR analysis for the capacity to bind the FGF8b sensor chip and the affinity of interaction (K_d_, mean ± SEM) was calculated for each peptide. nb, no binding. A schematic representation of ARPCA peptide is shown on the right. **C.** Overlay of selected region of 2D ^1^H-^13^C HSCQ NMR spectra. The spectral region of Hα-Cα correlations of A1 and A5 is reported in the absence of FGF8b (black) and at 0.5:1 (blue) and 1:1 (red) FGF8b:ARPCA ratios. **D.** Inhibition of HSPG/FGF8b/FGFR1 ternary complex formation by ARPCA. Data are the mean ± SEM of three determinations. **P < 0.01; ^#^*P* < 0.001.

The capacity of ARPCA to bind FGF8b was assessed by surface plasmonic resonance (SPR) spectroscopy [20]. To this purpose, increasing concentrations of the peptide were injected over a BIAcore sensor chip coated with the immobilized growth factor. As shown in Figure 1A, ARPCA binds to immobilized FGF8b in a dose-dependent manner with a *K*_d_ value equal to 278 ± 120 μM. On this basis, to assess the role of each ARPCA amino acid residue for FGF8b interaction, a series of synthetic peptides harboring different amino acid substitutions were assessed for their FGF8b binding activity by SPR analysis on the FGF8b-coated sensor chip.

As shown in Figure 1B, the partially scrambled Ac-APCRA-NH_2_ peptide did not show any significant inhibitory activity in this assay, pointing to the relevance of the relative position of RPC residues for the FGF8b antagonist capacity of the peptide. The activity was lost also when the Pro3 residue was replaced in Ac-ARACA-NH_2_ and Ac-ARGCA-NH_2_ peptides or when the Cys4 residue was replaced in Ac-ARPSA-NH_2_, Ac-ARPMA-NH_2_, and Ac-ARPVA-NH_2_ peptides, thus underlying the role of the RPC amino acid sequence in ARPCA/FGF8b interaction. Interestingly, as observed for ARPCA/FGF2 interaction [20], the FGF8b binding activity was dramatically reduced for the non-acetylated H-ARPCA-NH_2_ peptide and for the Ac-ARPCG-NH_2_ and Ac-GRPCG-NH_2_ peptides, indicating a role for the N-terminal blocking methyl group and for the methyl group of the side-chain of Ala1 and Ala5 residues in FGF8b interaction.

In order to characterize the structural basis of ARPCA/FGF8b binding interactions, FGF8b was titrated into a solution of ARPCA peptide and a series of 2D ^13^C-^1^H HSQC NMR experiments were recorded to follow peptide resonances. Progressive chemical shift variations were observed for Hα signals of Ala1 and Ala5 residues of the pentapeptide (Figure 1C), indicating that ARPCA makes specific interactions with FGF8b, mainly mediated by the two Ala residues. The titration progress was indicative of an intermediate to fast exchange phenomenon, pointing to a binding in the micromolar range, in good agreement with SPR data. Taken together, these results identify the ARPCA pentapeptide as a FGF8b binder. On this basis, ARPCA was further characterized for its capacity to interact with FGF8b and to antagonize its biological activity. The Ac-ARPVA-NH_2_ pentapeptide (hereafter named ARPVA) was used as a negative control.

FGFs exert their biological activity by leading to the formation of productive ternary complexes with signalling FGFRs and cell-surface heparan sulphate proteoglycans (HSPGs) [23]. On this basis, to investigate its FGF8b antagonist potential, ARPCA was evaluated for the capacity to prevent the formation of HSPG/FGF8b/FGFR1 ternary complexes in a cell-cell adhesion model in which FGF8b mediates the adhesion of FGFR1(III)c-overexpressing HSPG-deficient CHO cells to a HSPG-bearing CHO cell monolayer [16]. As shown in Figure 1D, ARPCA, but not ARPVA, exerts a dose-dependent inhibitory activity on FGF8b-mediated cell-cell adhesion (ID_50_ ~10 μM), thus indicating the capacity of the peptide to interfere with HSPG/FGF8b/FGFR1 complex formation.

### ARPCA inhibits the angiogenic activity of FGF8b

**Figure 2:**
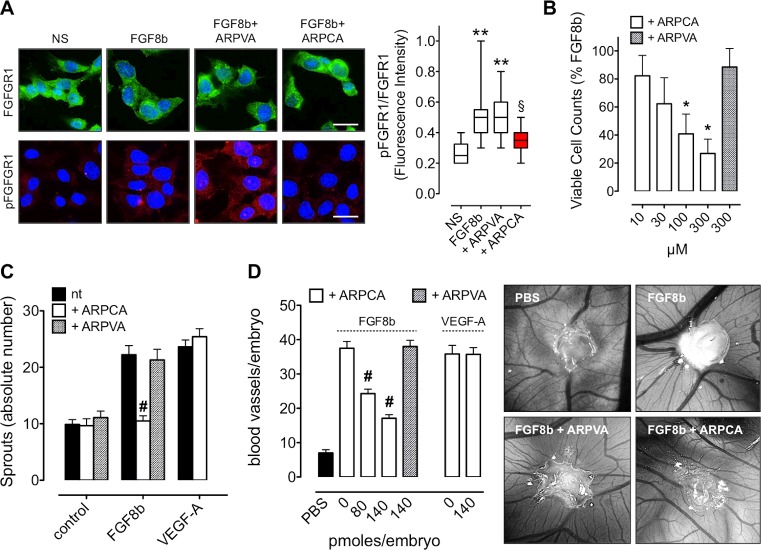
ARPCA inhibits the angiogenic activity of FGF8b **A.** HUVECs were stimulated with 30 ng/ml FGF8b in the presence of 60 μM ARPCA or ARPVA and immunostained with anti-FGFR1 (green) or anti-pFGFR1 (red) antibodies. Scale bar: 30 μm. Intensity of pFGFR1/FGFR1 signal was quantified and normalized to DAPI area (DAPI is in blue). The boxes extend from the 25th to the 75th percentiles, the lines indicate the median values, and the whiskers indicate the range of values. NS= not stimulated. **B.** Viable cell counting of HUVECs treated for 48 h with ARPCA or ARPVA in the presence of 30 ng/ml FGF8b. **C.** HUVEC spheroids were embedded in fibrin gel and treated with 30 ng/ml FGF8b or VEGF-A in the absence or presence of 60 μM ARPCA or ARPVA. After 24 h of stimulation the number of HUVEC sprouts were counted. **D.** Alginate pellets containing 4.5 pmoles of FGF8b or VEGF-A in the absence or presence of the indicated doses of ARPCA or ARPVA were placed on the top of the chick embryo CAM at day 11 of incubation. At day 14 newly formed blood vessels converging towards the implants were counted (8 embryos/group). Representative images of CAMs treated with FGF8b in the absence or presence of 140 pmoles of ARPCA or ARPVA are shown on the right. Data are the mean ± SEM. **P < 0.01 *vs* NS; §*P* < 0.05 *vs* FGF8b and +ARPVA; *P < 0.05; ^#^*P* < 0.001.

FGF8 acts on endothelial cells leading to the activation of the angiogenic process *in vitro* and *in vivo* [21]. In a first set of experiments, the capacity of ARPCA to impair the pro-angiogenic activity of FGF8b was assessed *in vitro* on human umbilical vein endothelial cells (HUVECs). In keeping with its capacity to prevent the formation of signaling HSPG/FGF8b/FGFR complexes, ARPCA, but not ARPVA, efficiently impairs FGFR1 phosphorylation triggered by FGF8b in HUVECs (Figure 2A). Accordingly, ARPCA inhibits HUVEC proliferation in response to FGF8b with an IC_50_ value equal to ~30 μM whereas no significant effect was observed when cells were treated with ARPVA at concentrations as high as 300 μM (Figure 2B). Also, ARPCA specifically prevents the pro-angiogenic/sprouting activity exerted by FGF8b on HUVEC spheroids embedded in fibrin gel with no effect on the angiogenic potential of vascular endothelial growth factor-A (VEGF-A), thus supporting the FGF-restricted specificity of the inhibitory effect (Figure 2C).

Finally, the capacity of ARPCA to affect FGF8b-induced neovascularization was investigated *in vivo* in a chick embryo chorioallantoic membrane (CAM) assay [24]. In this assay, alginate beads adsorbed with FGF8b (4.5 pmoles/plug) induce a potent angiogenic response when compared to control beads adsorbed with vehicle (Figure 2D). In keeping with the *in vitro* observations, the angiogenic response elicited by FGF8b was significantly reduced by the addition of 80 or 140 pmoles of ARPCA to the FGF8b implants (*P* < 0.0001). No inhibitory effect was instead exerted by control ARPVA or when ARPCA was challenged in the presence of VEGF-A as a pro-angiogenic stimulus (Figure 2D).

### ARPCA inhibits the proliferation and angiogenic potential of FGF8b-dependent tumor cells

**Figure 3:**
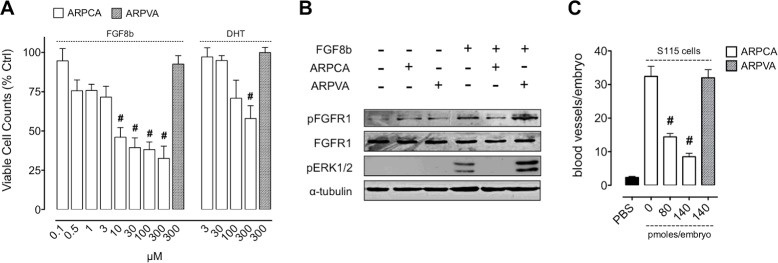
ARPCA inhibits the proliferation and angiogenic potential of FGF8b-dependent tumor cells **A.** S115 cells were treated with ARPCA or ARPVA in the presence of 30 ng/ml FGF8b or 10 nM DHT. Viable cells were counted 48 h thereafter. **B.** Western blot analysis of S115 cells treated with 30 ng/ml FGF8b in the absence or presence of 100 μM ARPCA or ARPVA. **C.** Alginate beads containing 2.5 × 10^4^ DHT-treated S115 cells were grafted onto the CAM at day 11 of incubation in the absence or presence of the indicated doses of ARPCA or ARPVA. At day 14, newly formed blood vessels were counted (8 embryos/group). Data are the mean ± SEM. ^#^*P* < 0.001.

Shionogi-115 (S115) cells represent a well-characterized murine model of androgen-regulated mammary tumor in which testosterone induces FGF8b upregulation that, in turn, increases FGFR1 expression, thus activating an autocrine loop of stimulation [16, 21]. As shown in Figure 3A, ARPCA impairs the mitogenic response of S115 cells to FGF8b or dihydrotestosterone (DHT) whereas ARPVA was ineffective. Accordingly, ARPCA, but not ARPVA, inhibits FGFR1 phosphorylation and downstream mitogen-activated protein kinase (MAPK) ERK_1/2_ activation in S115 cells treated with FGF8b (Figure 3B).

FGF8b produced by steroid hormone-regulated cancers promotes angiogenesis that sustains tumor growth *in vivo* [21]. To assess the effect of ARPCA on the angiogenic potential of FGF8b-producing tumor cells, S115 cells were incubated for 24 h with 10 nM DHT and then grafted onto the chick embryo CAM. As shown in Figure 3C, DHT-pretreated cells elicited a potent neovascular response that was abolished when cells were grafted in the presence of ARPCA at 80 or 140 pmoles/embryo (*P* < 0.001), no effect being exerted by control ARPVA. Together, these data indicate that ARPCA impairs the FGF8b-dependent mitogenic and angiogenic response triggered by DHT/FGF8b in S115 cells.

### ARPCA inhibits the *in vivo* growth of steroid hormone-regulated tumors

In order to assess the effect of ARPCA on the early phases of growth of FGF8b-dependent tumor grafts, S115 cells encapsulated in sodium alginate gel were injected s.c. in the flank of adult athymic *Nu/Nu* male mice. Then, mice were treated i.p. with 100 mg/kg of ARPCA or ARPVA on days 9, 11 and 13 after tumor challenge. On day 14 alginate plugs were harvested and processed for immunohistochemical analysis.

**Figure 4:**
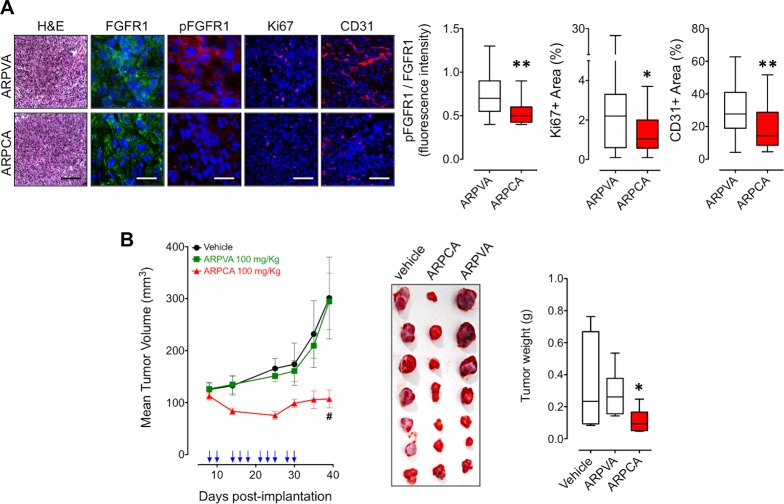
ARPCA inhibits the *in vivo* growth FGF8b/DHT-regulated S115 tumors **A.** Athymic male mice were implanted s.c. with alginate plugs containing S115 cells and treated i.p. every other day with 100 mg/kg of ARPCA or ARPVA (6 mice/group). After one week of treatment, plugs were harvested and processed for FGFR1, pFGFR1, Ki67 and CD31 immunofluorescence analysis. Scale bars: 30 μm (FGFR1 and pFGFR1) and 100 μm (H&E, Ki67, CD31). Intensity of pFGFR1/FGFR1 signal and Ki67^+^ or CD31^+^ areas were quantified and normalized to DAPI area (DAPI is in blue). **B.** Long-term tumor growth of S115 cells grafted s.c. in athymic male mice treated i.p. with vehicle or 100 mg/kg ARPCA or ARPVA. Treatments are indicated by arrows. At the end of the experiment, tumors were harvested, photographed and weighted (10-12 mice/group). Data are the mean ± SEM; *P < 0.05, **P < 0.01, ^#^*P* < 0.001.

As shown in Figure 4A, treatment with ARPCA efficiently inhibited FGFR1 phosphorylation, Ki67^+^ proliferation index and CD31^+^ neovascularization in tumor grafts when compared to lesions harvested from ARPVA-treated animals, thus confirming the inhibitory effect of ARPCA on *in vivo* S115 cell growth and angiogenic potential. On this basis, we performed a long-term experiment in which *Nu/Nu* male mice were injected s.c. with S115 cells and treated i.p. every other day with 100 mg/kg of ARPCA starting at day 8, when tumors were palpable. Tumor growth was followed for the next 32 days when tumors were harvested and weighted. The results demonstrate that ARPCA, but not ARPVA, suppresses S115 tumor growth (Figure 4B).

**Figure 5:**
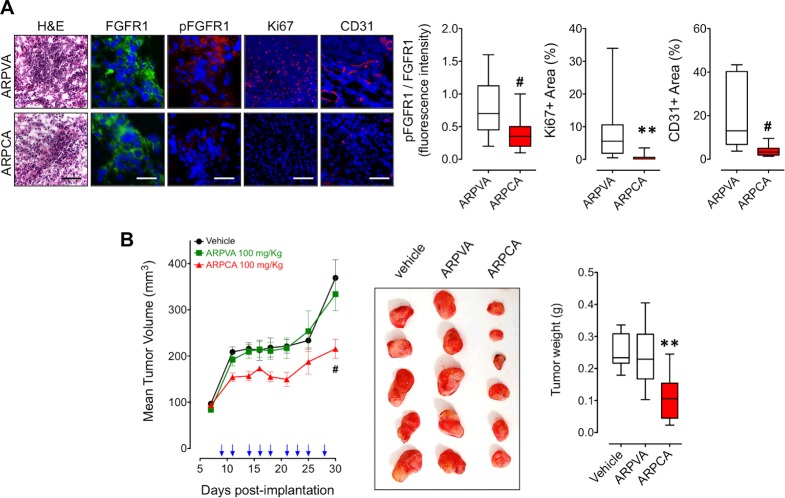
ARPCA inhibits the *in vivo* growth of FGF8b/FGF2/DHT-regulated TRAMP-C2 tumors **A.** C57BL/6 male mice were implanted s.c. with alginate plugs containing TRAMP-C2 cells and treated i.p. every other day with 100 mg/kg of ARPCA or ARPVA (6 mice/group). After one week of treatment, plugs were harvested and processed for FGFR1, pFGFR1, Ki67 and CD31 immunofluorescence analysis. Scale bars: 30 μm (FGFR1 and pFGFR1) and 100 μm (H&E, Ki67, CD31). Intensity of pFGFR1/FGFR1 signal and Ki67^+^ or CD31^+^ areas were quantified and normalized to DAPI area (DAPI is in blue). **B.** Long-term tumor growth of TRAMP-C2 cells grafted s.c. in C57BL/6 male mice treated i.p. with vehicle or 100 mg/kg ARPCA or ARPVA. Treatments are indicated by arrows. At the end of the experiment, tumors were harvested, photographed and weighted (10-12 mice/group). Data are the mean ± SEM; ** < 0.01, ^#^*P* < 0.001.

**Figure 6:**
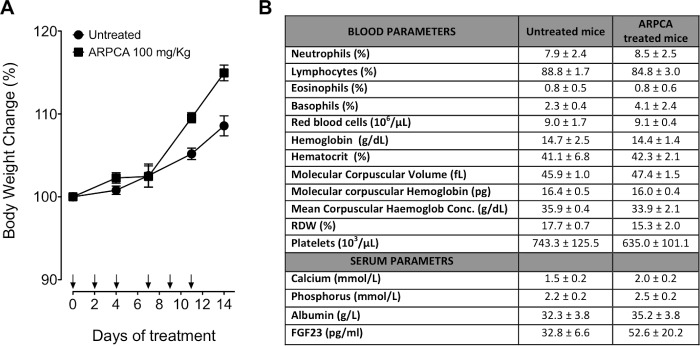
Body weight variation and hematological parameters of mice after treatment with ARPCA **A.** C57BL/6 mice were treated i.p. every other day for two weeks with ARPCA at 100 mg/kg (arrows). At different time points, the percentage of body weight variation was calculated in respect to Day 0. B) At the end of ARPCA treatment (Day 14), blood components, biochemical serum parameters and FGF23 serum levels were determined. Data are the mean ± standard deviation of 3 or more animals.

Previous observations had shown that FGF8b and FGF2 play a key role in prostate cancer and that PTX3 overexpression inhibits the FGF8b/FGF2-driven growth of TRAMP-C2 tumors [15], an androgen-responsive murine model of prostate carcinoma [22]. Given the capacity of ARPCA to act as a FGF2/FGF8b-trap ([20] and present work), we assessed its therapeutic potential also in this steroid-regulated tumor model driven by the autocrine/paracrine action of both FGFs. To this purpose, TRAMP-C2 cells were embedded in alginate plugs and injected s.c. in syngeneic C57BL/6 male mice. As observed for early S115 lesions, treatment with ARPCA (i.p. at 100 mg/kg every other day) impaired FGFR1 activation in TRAMP-C2 alginate plugs, thus leading to a decrease of the tumor cell proliferation rate (as assessed by Ki67 immunostaining) and of CD31-positive tumor vascularization (Figure 5A). Accordingly, ARPCA, but not ARPVA, inhibited the growth of TRAMP-C2 tumor grafts in a long-term assay (Figure 5B). Of note, long-term administration of ARPCA did not affect body weight, hematologic parameters, blood serum components and FGF23 serum levels in treated animals (Figure 6).

## DISCUSSION

FGF8b affects epithelial/stromal compartments of steroid hormone-regulated tumors by exerting an autocrine activity on cancer cells and a paracrine pro-angiogenic function that may contribute to tumor progression [4-6, 8]. Indeed, FGF8b is overexpressed in a high proportion of human breast [25] and aggressive prostate cancers [26]. Also, FGF8b plays a non-redundant role in the activation of FGFR signaling cascade that confers resistance to hormone deprivation therapy and drives the progression toward a hormone-independent neoplasm [27]. On this basis, the FGF/FGFR system has been hypothesized as a target for the treatment of steroid hormone-regulated tumors [4, 27, 28], also in a possible synergistic combination with radiotherapy or classical chemotherapy [29].

Previous observations had shown that the soluble pattern recognition receptor PTX3 binds FGF8b and inhibits the angiogenic and tumorigenic activity of androgen-regulated tumor cells in which testosterone activates a FGF8b-dependent autocrine/paracrine loop of stimulation [15]. PTX3 interacts with different ligands *via* its N-terminal or C-terminal domain [12, 17]. An integrated approach that utilized recombinant N-terminal and C-terminal PTX3 fragments, monoclonal antibodies, and SPR analysis identified the FGF2-binding domain in the PTX3 N-terminus [14]. Moreover, as observed for FGF2 [13, 14], a free recombinant N-terminal PTX3 fragment prevented the binding of FGF8b to immobilized PTX3 [16], thus implicating the N-terminal PTX3 extension also in this interaction.

Here, we demonstrate that the pentapeptide ARPCA, corresponding to the amino acid sequence 100-104 in PTX3 N-terminus, represents a novel FGF8b antagonist endowed with antiangiogenic and antineoplastic activity. Indeed, ARPCA binds FGF8b, thus preventing the formation of signalling HSPG/FGF8b/FGFR1 ternary complexes. Accordingly, ARPCA inhibits the angiogenic activity exerted *in vitro* and *in vivo* by FGF8b. Also, ARPCA inhibits the proliferation and angiogenic potential of androgen-regulated murine mammary S115 tumor cells driven by FGF8b as well as by testosterone. As a result of its ability to inhibit both paracrine and autocrine functions of FGF8b, ARPCA exerts a significant inhibitory effect on FGFR1 phosphorylation, cell proliferation, angiogenic and tumorigenic activity of S115 tumor grafts in immunodeficient male mice. Furthermore, ARPCA inhibits the FGF8b/FGF2-driven growth and vascularization of TRAMP-C2 tumors, an androgen-responsive murine model of prostate carcinoma [22].

Notably, the anti-tumor action of ARPCA occurred in the absence of any systemic toxic effect in treated animals. In particular, at variance with other inhibitors of the FGF/FGFR system [30], the safety profile of ARPCA treatment included the absence of any effect on the blood levels of FGF23, calcium and phosphorus. Accordingly, SPR analysis did not show any interaction of ARPCA with immobilized FGF23 (data not shown).

VEGF plays a central role in tumor neovascularization and inhibition of the VEGF/VEGF receptor system markedly disrupts angiogenic switching and initial tumor growth. However, targeting FGFs in addition to VEGF might show synergistic effects in the treatment of angiogenesis-dependent diseases, including cancer [1, 31]. Also, experimental evidences indicate that drug resistance to VEGF blockade may occur following reactivation of the angiogenic process triggered by the compensatory upregulation of the FGF/FGFR system in experimental tumor models [32] and in cancer patients [33], representing a mechanism of escape to anti-VEGF therapy in cancer treatment [31]. Our preclinical observations support the notion that inhibition of FGF8b activity by ARPCA suppresses the initial phases of growth and vascularization of androgen-regulated tumors, thus resulting in the inhibition of tumor progression in long-term tumor assays. This occurs despite the fact that ARPCA does not affect the pro-angiogenic action of VEGF-A and may be related to its ability to affect both tumor epithelial and stromal compartments by suppressing the autocrine/paracrine action of FGF8b that is essential for the initial angiogenic and proliferative switch of tumor cells.

Various amino acid substitutions in the ARPCA sequence, including the removal of the N-terminal blocking acetyl group, cause a dramatic decrease in the FGF8b-binding capacity of the corresponding mutated synthetic pentapeptides, pointing to the relevance of each amino acid residue for ARPCA/FGF8b interaction. NMR experiments demonstrate that Ala1 and Ala5 make direct contacts with the FGF8b protein and the involvement of their methyl groups in FGF8b interaction is supported by the observed lack of activity of the Ac-GRPCG-NH_2_ mutant. On the other hand, the RPC sequence plays a conformational role in ARPCA/FGF8b interaction and may help to orient the methyl groups of the peptide for optimal interaction with the growth factor. This hypothesis is supported by the lack of FGF8b-binding activity of Pro3 or Cys4 peptide mutants and of the partially scrambled Ac-APCRA-NH_2_ peptide.

Hydrophobic interactions are implicated in ARPCA binding to FGF8b. X-ray crystallography data have shown that hydrophobic interactions dominate the interface between FGFs and the D2 domain of the FGFR extracellular moiety [34, 35]. On this basis, ARPCA likely exerts its FGF antagonist activity by mimicking the hydrophobic region of D2, thus competing with FGFRs for the binding to the growth factor, as hypothesized for ARPCA/FGF2 interaction [20]. Indeed, ARPCA hampers the capacity of FGF8b to form FGFR1-mediated ternary complexes with HSPGs, thus inhibiting FGFR1 activation and signalling triggered by FGF8b in endothelial and cancer cells. It must be pointed out that the unique spatial positioning of the FGF8b N-terminal g-helix allows for a hydrophobic contact also with the groove in the FGFR D3 domain [35], thus representing a possible alternative/additional ARPCA interaction site for FGF8b (Supplemental Figure S1). Further studies will be required to unambiguously define the molecular bases of ARPCA/FGF8b interaction.

Previous findings had shown that ARPCA is endowed with a potent anti-FGF2 activity *in vitro* and *in vivo* [20]. Here, we demonstrate the ability of ARPCA to antagonize also the pro-angiogenic and tumorigenic activity of FGF8b. Accordingly, ARPCA exerts a significant inhibitory on the growth and vascularization of TRAMP-C2 tumor grafts, a classical model of steroid hormone-dependent prostate cancer characterized by FGF2/FGF8b co-expression. Thus, ARPCA may act as a FGF2/FGF8b antagonist able to affect tumor epithelial and stromal compartments by suppressing the autocrine/paracrine action of both growth factors. Relevant to this point, SPR experiments indicate that ARPCA is able to interact also with other members of the FGF family, including FGF1, FGF5, FGF7, FGF16, FGF17, FGF18, FGF20 and FGF22 (Table 1). All these FGFs have been shown to play a role in different human cancers [36-42]. Experiments are in progress to translate the information about ARPCA/FGF interaction into a pharmacophore model to be used for the screening of small molecule databases [43], in the search for a novel low molecular weight multi-FGF trap for the therapy of FGF-driven cancers.

**Table 1:** Surface plasmon resonance (SPR) analysis of FGF/ARPCA interaction

FGFs	K_d_ (μM) (mean ± SEM)
**FGF1 subfamily**	
FGF1	227 ± 46
FGF2	1700 ± 100
**FGF4 subfamily**	
FGF4	n.b.
FGF5	299±160
FGF6	n.b.
**FGF7 subfamily**	
FGF3	n.b.
FGF7	885 ± 240
FGF10	n.b.
FGF22	129 ± 57
**FGF8 subfamily**	
FGF8b	278 ± 120
FGF17	476 ± 265
FGF18	26 ± 4
**FGF9 subfamily**	
FGF9	n.b.
FGF16	112 ± 23
FGF20	264 ± 97

ARPCA does not affect the pro-angiogenic action of VEGF-A and may be related to its ability to affect both tumor epithelial and stromal compartments by suppressing the autocrine/paracrine action of FGF8b that is essential for the initial angiogenic and proliferative switch of tumor cells.

## MATERIALS AND METHODS

### Reagents and cell culture

Human recombinant FGF8b was from PeproTech, ARPCA, ARPVA and scrambled peptides were kindly provided by R. Longhi and A. Gori (CNR, Milano, Italy). Recombinant FGF8b for NMR studies was produced and purified by ASLA (Riga, Latvia. EU). Alginic acid sodium salt was from Sigma-Aldrich (Saint Louis, MO, USA).

HUVECs were used at passages I–IV and grown on plastic surface coated with porcine gelatin (Sigma-Aldrich) in M199 medium (Invitrogen, Carlsbad, CA, USA) supplemented with 20% fetal bovine serum (FBS) (Invitrogen), endothelial cell growth factor (100 μg/mL) (Sigma-Aldrich), and porcine heparin (100 μg/mL, Sigma-Aldrich). S115 mouse mammary carcinoma cells were kindly provided by M. Jalkanen (Biotie, Turku, Finland) and maintained in DMEM supplemented with 5% heat-inactivated FBS, 1 mM sodium pyruvate, 1 mM glutamine and 10 mM testosterone (DHT). Murine prostate adenocarcinoma TRAMP-C2 cells were obtained from ATCC repository and maintained in DMEM supplemented with 10% heat inactivated FBS, 10 mM HEPES buffer, 0.5 mM 2-mercaptoethanol, 2.0 mM glutamine, 5 mg/ml bovine insulin (Sigma-Aldrich) and 10 nM DHT. Cells were maintained at low passage, returning to original frozen stocks every 3 to 4 months, and tested regularly for *Mycoplasma* negativity.

### Surface Plasmon Resonance (SPR) analyses

A BIAcore X-100 apparatus (BIAcore Inc., Piscataway, NJ, USA) was used to set up the following experimental models. ARPCA and all the peptides mentioned in the present work were analyzed for their capacity to directly bind to immobilized FGF8b. To this purpose, FGF8b (20 μg/ml in 10 mM sodium acetate, pH 6.0) was allowed to react with a flow cell of a CM5 sensor chip that was previously activated with a mixture of 0.2 M N-ethyl-N′-(3-dimethylaminopropyl)-carbodiimide hydrochloride and 0.05 M N-hydroxysuccinimide (35 μl, flow rate 10 μl/min). After ligand immobilization, matrix neutralization was performed with 1.0 M ethanolamine (pH 8.5) (35 μl, flow rate 10 μl/min) and the activated/deactivated dextran was used as reference (control) system. Increasing concentrations of ARPCA (ranging between 8 μM and 2000 μM) were injected over the FGF8b-coated sensor chip and the response was recorded as a function of time tracking the SPR intensity change upon binding progression. Injection lasted for 4 min (flow rate 30 μl/min) to allow peptide association to immobilized FGF8b and was followed by 10 min of dissociation; each run was performed in HBS and the sensor chip was regenerated with 10 mM NaOH. The equilibrium (plateau) values of the SPR sensorgrams were used to build the binding isotherms (dose-response curves) displayed. Binding isotherm points were fitted with the Langmuir equation for monovalent binding. This allowed to evaluate the mass surface dissociation constant, *K*_d_, and the scaling parameter that relates the SPR signal with the extent of binding, as the free parameters of the fitting. The errors on these parameters were assigned as a result of the fitting algorithm (95% confidence bounds). The best-fitting procedure was performed with the SigmaPlot 11.0 software package (Systat Software Inc.). Others FGFs were dissolved in 10mM sodium acetate at optimal pH, immobilized on CM5 sensorchips and tested for their capacity to bind ARPCA as described above.

### ARPCA-FGF8 interactions assessed by NMR

NMR experiments were collected on samples containing 190 μM of ARPCA, dissolved in 50 mM potassium phosphate buffer pH 6.7 (90% H2O/10% D2O), 2.5 mM arginine, 10mM NaCl in the presence of variable concentrations of FGF8 corresponding to 1:0, 1:0.5, and 1:1 ARPCA:FGF8 ratios.

2D 1H-13C HSQC spectra were recorded with a sweep width of 10 ppm and 100 ppm.2k × 128 data points were used in proton and carbon dimensions, respectively. Data, acquired and processed using Topspin (Bruker Biospin), were apodized with a squared sinebell shifted by 90° and polynomial baseline correction. All NMR spectra were recorded at 298 K with a Bruker DMX spectrometer operating at 600 MHz.

### HSPG/FGF8b/FGFR1 mediated cell-cell adhesion assay

This assay was performed as described [44] with minor modifications. Briefly, wild-type CHO-K1 cells were seeded in 24-well plates at 150000 cells/cm^2^. After 24 h, cell monolayers were washed with PBS and incubated with 3% glutaraldehyde in PBS for 2 h at 4°C. Fixation was stopped with 0.1 M glycine and cells were washed extensively with PBS. Then, A745-CHO-flg-1A-luc cells (50000 cells/cm^2^) were added to CHO-K1 monolayers in serum-free medium *plus* 10 mM EDTA with or without 30 ng/ml FGF8b in the absence or presence of increasing concentrations of the ARPCA or ARPVA peptides. After 2 h of incubation at 37°C, unattached cells were removed by washing twice with PBS, and A745-CHO-flg-1A-luc bound to the CHO-K1 monolayer were solubilized and luciferase activity was quantified. All experiments were performed in triplicate.

### Cell proliferation assays

HUVECs were seeded at 18000 cells/cm^2^ in medium containing 2.5% FBS and stimulated with 30 ng/ml FGF8b in the absence or presence of increasing concentrations of ARPCA or ARPVA. S115 cells were seeded at 15000 cells/cm^2^ in medium containing 4% hormone-deprived dextran-coated charcoal-stripped heat inactivated FBS, followed by 24 h serum starvation in 1:1 mixture of serum-free Ham's F12 and DMEM. Cells were stimulated for 48 h with 30 ng/ml FGF8b or 10 nM DHT in the presence or absence of different concentrations of ARPCA or ARPVA (as specified in the corresponding figures). Viable cell counts were obtained by the counting function of the MACSQuant^®^ Analyzer (Miltenyi Biotec, Bergisch-Gladbach, Germany, EU).

### Endothelial cell sprouting assay

HUVEC spheroid aggregates were prepared in 20% methylcellulose medium, embedded in fibrin gel, and stimulated with FGF8b or VEGF-A (both at 30 ng/ml) plus 5% FBS in the absence or presence of 60 μM ARPCA or ARPVA. Formation of radially growing cell sprouts was observed during the next 24 h, photographed at 200x magnification using an Axiovert 200M microscope (Carl Zeiss, Milan, Italy, EU) and counted.

### Western blotting

S115 cells were treated with FGF8b (30ng/ml) in the presence or absence of ARPCA (100 μM) or ARPVA (100 μM). After 20 minutes of incubation cell samples were washed in cold PBS and homogenized in RIPA buffer containing 1% Triton-X100, 0.2% BriJ, 1 mM sodium orthovanadate and protease inhibitors cocktail. Protein concentrations were determined using the Bradford protein assay (Bio-Rad Laboratories, Milano, Italy). Blotting analysis was performed using anti-FGFR1, anti-phospho FGFR1 and anti-phosphoERK_1/2_ antibodies (Santa Cruz Biotechnology, Santa Cruz, CA, USA). Equal loading of the lanes was confirmed by immunoblotting with anti–αTubulin (Sigma-Aldrich) antibody.

### *In vitro* immunofluorescence analysis

HUVECs were seeded in Ibidi^®^ μ-Slide 8 wells (Ibidi, Martinsried, Germany, EU) at a density of 30000 cells/cm^2^, starved in 2.5% FBS for 24 h and stimulated for 20 minutes with 30 ng/ml of FGF8b in the absence or presence of ARPCA or ARPVA. Following stimulation, cells were washed twice in PBS, fixed in cold acetone for 5 minutes and permeabilized with 0.2% Triton-X100 in PBS for 2 minutes at room temperature (RT). After washing in PBS, cells were blocked for 10 minutes at RT in 1% BSA and then incubated with rabbit anti-pFGFR1 or rabbit anti-FGFR1 antibodies (Santa Cruz Biotechnology) for 1 h at RT. After washing in PBS, cells were incubated with AlexaFluor 594-conjugated anti-rabbit antibody (Invitrogen) and DAPI for 30 minutes at RT. Finally, cells were examined under a Zeiss Fluorescence Axiovert 200M microscope (Carl Zeiss).

### Chick embryo chorioallantoic membrane (CAM) assay

Alginate plugs containing vehicle, FGF8b (4.5 pmoles/embryo), VEGF-A (4.5 pmoles/embryo) or S115 cells (2.5×10^4^/embryo) in the absence or presence of ARPCA (80 or 140 pmoles/embryo) or ARPVA (140 pmoles/embryo) were placed on the CAM of fertilized white leghorn chicken eggs at day 11 of incubation. At day 14, newly formed blood microvessels converging toward the implant were counted by two observers in a double-blind fashion at 5x magnification under a stereomicroscope (STEMI-SR, x2/0.12; Zeiss) as described [24].

### Murine s.c. alginate implant assay

Eightweek-old athymic *Nu/Nu* or C57BL/6 male mice were injected s.c. with 450 μl of 3% (w/v) sodium alginate solution (alginic acid dissolved in LPS-free PBS) containing 5×10^6^ S115 or 8×10^6^ TRAMP-C2 cells, respectively [15]. Mice were treated i.p. with ARPCA or ARPVA (100 mg/kg) on day 7, 9, 11 and 13 after implant. On day 14, alginate gels were harvested, weighted, embedded in OCT-compound and immediately frozen to be processed for histological analysis. For immunofluorescence stainings, sections (5 μm thick) were obtained with a cryostat microtome, air dried and fixed with acetone (for 5 min at 4°C). After blocking with 1% BSA (Bovine Serum Albumin) in PBS for 10 minutes, sections were incubated with primary antibodies, rabbit anti-FGFR1 (Santa Cruz Biotechnology), rabbit anti-phospho FGFR1 (Santa Cruz Biotechnology), Ki67 (Dako, Milano, Italy, EU) or CD31 (BD Biosciences Pharmingen, San Diego, CA, USA). After washing with PBS containing 0.05% Tween 20, sections were incubated with the appropriate Alexa Fluor 488 or Alexa Fluor 594-conjugated secondary antibody (Invitrogen). After mounting in a drop of anti-bleaching mounting medium containing DAPI (Vectashield, Vector Laboratories, Burlingame, CA, USA), sections were examined under a Zeiss Fluorescence Axiovert 200M microscope (Carl Zeiss).

### *In vivo* tumor therapy studies

Eight week old C57BL/6 male mice were injected s.c. with 8×10^6^ TRAMP-C2 cells, and eight week old athymic *Nu/Nu* male mice were injected s.c. with 5×10^6^ S115 cells. All injections were performed in 200 μl total volume of PBS into the dorsolateral flank. When tumors were palpable treatment was performed every other day by i.p. injection of ARPCA, ARPVA (both at 100 mg/kg) or vehicle (PBS) in 100 μl final volume. Tumors were measured in two dimensions and tumor volume was calculated according to the formula V=(D × d^2^)/2, where D and d are the major and minor perpendicular tumor diameters, respectively [45]. At the end of the experimental procedure tumors were harvested, weighted and photographed.

### *In vivo* ARPCA toxicity studies

Eight week-old C57BL/6 mice were treated i.p. with 100 mg/kg of ARPCA every other day for two weeks. During this period, animals were weighted for body weight variation analysis. At the end of treatment, whole blood and serum were harvested and analysed for blood components, biochemical serum parameters and circulating levels of FGF23. The serum levels of FGF23 were assessed by ELISA (Uscn Life Science Inc., Wuhan, PRC, Asia) according to manufacturer's instructions. Untreated mice were used as reference/control.

### Statistical analysis

Statistical analyses were performed using the statistical package Prism 5 (GraphPad Software). Student's t test for unpaired data (2-tailed) was used to test the probability of significant differences between two groups of samples. For more than two groups of samples, data were statistically analyzed with a 1-way analysis of variance, and individual group comparisons were evaluated by the Bonferroni multiple comparison test. Tumor volume data were statistically analyzed with a 2-way analysis of variance, and individual group comparisons were evaluated by the Bonferroni correction. Differences were considered significant when *P* < 0.05.

## SUPPLEMENTARY MATERIAL FIGURE


