# Supplementary material for: A long pentraxin-3-derived pentapeptide for the therapy of FGF8b-driven steroid hormone-regulated cancers
Source: Oncotarget. 2015 Apr 14;6(15):13790–802. doi: 10.18632/oncotarget.3831 (PMC4537050; doi:10.18632/oncotarget.3831)
Supplement: Supplementary file 1 [file oncotarget-06-13790-s001.pdf]

A long pentraxin-3-derived pentapeptide for the therapy of FGF8b-driven steroid hormone-regulated cancers

Supplementary Material

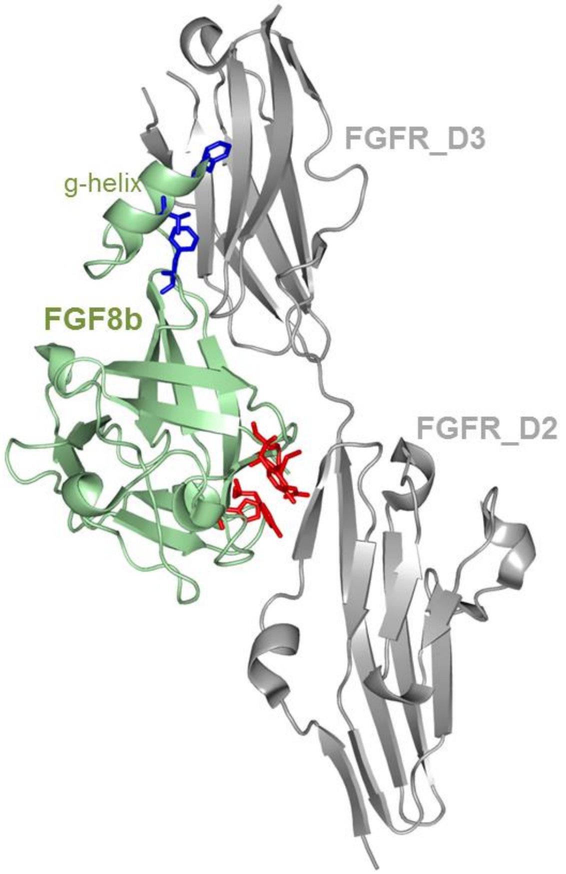

Supplemental Figure S1: Mode of FGF8b/FGFR interaction.

Residues belonging the FGF8b hydrophobic interfaces with FGFR2c_D2 and FGFR2c_D3 are highlighted on the FGF8b structure as red and blue sticks, respectively (PDB ID: 2FDB). FGF8b and FGFR domains are shown as green and grey cartoons, respectively, and the N-terminal g-helix FGF8b(32-40) is indicated.
